# Congenital extrahepatic portosystemic shunt (Abernethy malformation) treated with surgical shunt ligation: A case report and literature review

**DOI:** 10.1016/j.ijscr.2019.11.014

**Published:** 2019-11-20

**Authors:** Chollasak Thirapattaraphan, Suporn Treepongkaruna, Nichanan Ruangwattanapaisarn, Suthida Sae-Guay

**Affiliations:** a Division of Pediatric Surgery, Department of Surgery, Faculty of Medicine Ramathibodi Hospital, Mahidol University, 270 Rama VI Road, Tung Phayathai, Ratchathewi, Bangkok 10400, Thailand; b Division of Gastroenterology, Department of Paediatrics, Faculty of Medicine Ramathibodi Hospital, Mahidol University, 270 Rama VI Road, Tung Phayathai, Ratchathewi, Bangkok 10400, Thailand; c Department of Diagnostic and Therapeutic Radiology, Faculty of Medicine Ramathibodi Hospital, Mahidol University, 270 Rama VI Road, Tung Phayathai, Ratchathewi, Bangkok 10400, Thailand; d Division of Pediatric Surgery, Department of Surgery, Phramongkutklao Hospital, 315 Rajavithi Road, Tung Phayathai, Ratchathewi, Bangkok, 10400, Thailand

**Keywords:** Congenital extrahepatic portosystemic shunt, Abernethy malformation, Surgical shunt ligation

## Abstract

**Introduction:**

Congenital extrahepatic portosystemic shunt is also known as Abernethy malformation. It is a rare anomaly of the portal venous system where partial or complete portal blood flow drains directly into the systemic vein via this abnormal shunt, bypassing the liver. Occasionally, this condition is incidentally detected on imaging studies performed for other indications. An early diagnosis is challenging due to the lack of specific symptoms—often leading to delayed diagnosis and development of subsequent complications.

**Presentation of case:**

A 4-year-old boy presented with multiple cutaneous hemangiomas with concomitant splenomegaly. While the diagnostic work-up for the definite cause of splenomegaly was in progress, an Abernethy malformation was incidentally noted by abdominal ultrasonography. After computerized tomography (CT) imaging confirmed the diagnosis, the patient underwent a successful surgical shunt ligation without any complications through a one-year follow-up period.

**Discussion:**

Various imaging studies, including Doppler ultrasonography, CT imaging, and Magnetic Resonance Imaging (MRI), are useful tools to diagnose an Abernethy malformation. Preoperative angiography with a balloon occlusion of the shunt is highly recommended in order to opt for the most appropriate intervention—including one-or-two-step surgical closure, endovascular occlusion, and liver transplantation.

**Conclusion:**

An Abernethy malformation should be considered in the differential diagnosis of unexplained portal hypertension or any of its associated complications in pediatric patients. Early diagnosis and proper management of this condition can lead to a favorable prognosis, as in this case. Accordingly, a pre-operative evaluation is important to determine the proper therapeutic method.

## Highlights

• Abernethy malformation is a rare congenital abnormality characterized by an extrahepatic portosystemic shunt.
• Doppler ultrasonography is usually the initial investigation.
• Computerized tomography (CT) scan or magnetic resonance imaging (MRI) scan is helpful in confirming the diagnosis.
• Preoperative angiography with balloon occlusion test is highly recommended to determine the most appropriate intervention.
• We propose guidelines for approaching and management this condition.

## 1 Introduction

A congenital portosystemic shunt (CPSS) is a rare vascular malformation, consisting of an abnormal connection between portal and systemic veins [1]. The overall prevalence of CPSS is approximately 1 in 30,000 births, as reported in a galactosemic screening study of neonates [2]. The diagnosis is challenging to establish due to its rarity and non-specific symptoms. With recent advances in imaging modalities, some asymptomatic cases have been incidentally detected by clinical imaging performed for other indications. However, the diagnosis was often delayed in most of the cases, leading to subsequent complications including hepatopulmonary syndrome, hepatic encephalopathy, portal hypertension, and hepatic neoplasms [1].

To our knowledge, this is the first report of a congenital extrahepatic portosystemic shunt, also known as Abernethy malformation, in Thailand. Our objective was to report and clearly delineate the approaches and management of this rare case. This case report has been reported in accordance with the surgical case report (SCARE) guidelines [3].

## 2 Case presentation

**Fig. 1:**
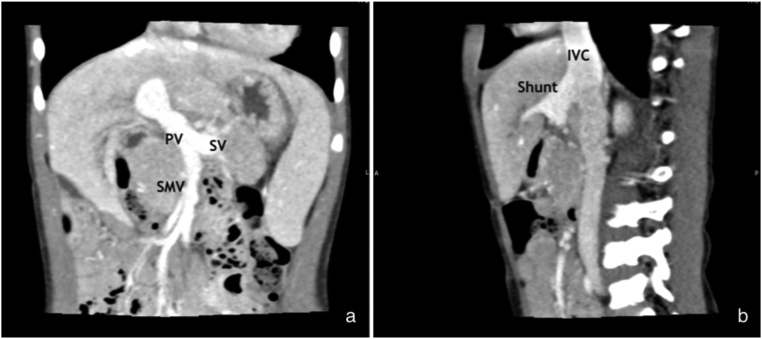
A contrast-enhanced CT scan showed the enlarged main portal vein (PV), splenic vein (SV), superior mesenteric vein (SMV), and a portosystemic shunt connecting the left portal vein to inferior vena cava (IVC).

A 4-year-old boy presented with multiple discrete subcutaneous reddish papules and nodules over the entirety of his body. Physical examination revealed splenomegaly and multiple cutaneous hemangiomas. Neither of jaundice, hepatomegaly nor abnormal ascites was noted. The cutaneous hemangiomas resolved nine months after treatment with oral propranolol. Initial blood tests revealed only an elevated serum ammonia level of 85 μg/dL (normal range, 15–51 μg/dL). Complete blood count (CBC) and liver function tests proved normal. In order to investigate the cause of his splenomegaly, Doppler ultrasonography initially detected an anomalous vessel in the liver. An Abdominal CT scan subsequently confirmed a portosystemic shunt connecting the left portal vein to the inferior vena cava (IVC). The Enlarged main portal, splenic and superior mesenteric veins were examined (Fig. 1). Also noted were a marked narrowing of the right portal vein and its peripheral branches without color flow. Thus, the patient was diagnosed with a portosystemic shunt or Abernethy malformation type II.

A cavoportogram via a trans-jugular access was performed, revealing a large portal-IVC shunt measuring 15 mm in diameter and 8 mm in length. However, the embolization with a vascular plug could not be done in this case due to an inadequate length of the affected vessels to place the device. Therefore, we decided to proceed with a surgical shunt ligation two weeks later. The intraoperative findings demonstrated a left portal vein-IVC shunt, measuring 15 mm in diameter—the same size as was evaluated with the cavoportogram. The hypoplastic right portal vein was 3 mm in diameter. The shunt was encircled with a vascular sling and temporarily clamped. The portal vein pressures were measured before and after procedures: 15 and 23 mmHg, respectively. The shunt was then completely ligated with a non-absorbable 1-0 silk suture.

**Fig. 2:**
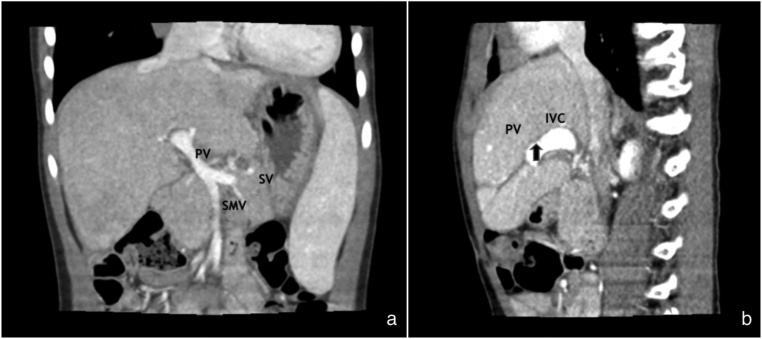
A contrast-enhanced CT scan at 6 months postoperatively showed disconnection between the left portal vein (PV) and IVC.

The patient had attended follow-up visits over a 12-month postoperative period. Doppler ultrasonography and CT imaging of the liver at 1 month, 2 months and 6 months showed a favorable resolution (Fig. 2). Additionally, his serum ammonia level had decreased to less than 15 μg/dL.

## 3 Discussion

CPSS is divided into two main categories as follows: (1) intrahepatic type including portohepatic shunt and patent ductus venosus (PDV), and (2) extrahepatic type or “Abernethy malformation”. The latter is named such because it was first described by John Abernethy in 1793. He reported a postmortem examination in a 10-month-old female infant, who showed multiple congenital anomalies including dextrocardia, transposition of great vessels, polysplenia, and termination of the portal vein in IVC at the level of the insertion of the renal vein with the enlarged hepatic artery [4]. Since then, “Abernethy malformation” has been used to describe the congenital diversion of portal blood away from the liver in recognition of the first accurate description of this condition [5]. Recently, there are many classifications of congenital portosystemic shunts as shown in Table 1.

**Table 1:** Classifications of congenital portosystemic shunts.

Classification author	Based on	Types
Morgan and Superina (1994) [6]	Intrahepatic blood flow via portal vein	Type I: Total shunt (Congenital absence of portal vein)
		- Ia: Superior mesenteric vein and splenic vein do not join to form confluence - Ib: Superior mesenteric vein and splenic vein join to form confluence
		Type II: Present portal vein with partial shunt - IIa: Congenital - IIb: Acquired
Lautz T et al. (2011) [7]	The origin of the shunt	Type I: No intrahepatic portal flow
		Type II: Partial shunt with preserved hepatic flow
		- IIa: Shunt arising from left or right portal vein (includes PDV) - IIb: Shunt arising from main portal vein (including its bifurcation or splenomesenteric confluence) - IIc: Shunt arising from mesenteric, gastric or splenic
Blanc et al. (2014) [8]	Caval ending of the shunt	1. Extrahepatic portosystemic shunt (EHPS) patterns 2. Porto-caval (PC) patterns 2.1 End-to-side-like PC (ESPC) 2.2 Side-to-side-like PC (SSPC) 2.3 H-type PC (HPC) 3. Portohepatic (PH) pattern 4. Persistent ductus venosus (PDV) pattern
Kanazawa et al. (2015) [9]	Hypoplasia of intrahepatic portal system (IHPS)	1. Mild type: well-visualized IHPS distributed with uniformity of periphery of the liver 2. Moderate type: a moderately visualized IHPS with appearance of a puff of smoke 3. Sever type: little or no visualized IHPS

Because of the non-specific clinical presentation, the imaging studies play an important role for diagnosis. Doppler ultrasonography is usually the initial investigation, and it is also useful in the follow-up period after treatment. CT or MR imaging is helpful in confirming the diagnosis, providing a vascular map, and searching for complications and its associated anomalies such as liver nodules. Angiography and portal pressure measurement while temporary occluding the shunt (shunt occlusion test) is a crucial procedure to evaluate the degree of hypoplasia presenting in the intrahepatic portal veins and to measure the portal pressure during shunt occlusion [1]. This diagnostic test is especially indicated in extrahepatic CPSS with end-to-side portocaval shunt or type I Abernethy malformation. Because, in certain patients, intrahepatic portal branches cannot be detected by abdominal ultrasonography or contrast-enhanced CT but they are merely visualized by angiography with shunt occlusion [9].

**Fig. 3:**
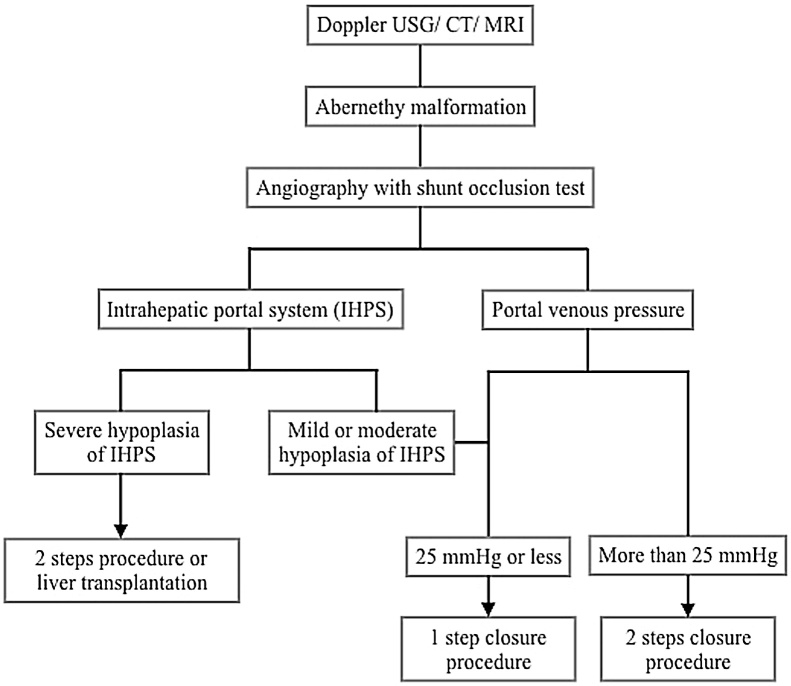
An algorithm for management of extrahepatic portosystemic shunts.

Closure of the extrahepatic CPSS should be considered early, especially in patients who present with associated complications. Because of its rarity, there is no standard guideline for the management of CPSS. We hereby propose an algorithm for the management of extrahepatic portosystemic shunts (Fig. 3). The therapeutic approach depends on the portal pressure and the condition of the intrahepatic vasculature determined by a balloon occlusion test. Nevertheless, there is no precise cut-off point of portal pressure for a 1-step closure. The most acceptable final portal pressure under shunt occlusion is not higher than 25 mmHg [7,9]. Otherwise, a 2-step procedure is recommended to avoid post-closure complications—including severe portal hypertension, bowel congestion, and bacterial translocation. The severity of the hypoplasia of IHPS is also a potential factor to determine the most appropriate therapeutic approach. According to the IHPS classification by Kanazawa et al. [9], all patients with a mild-to-moderate degree of hypoplasia and a portal pressure of less than 25 mmHg could be successfully treated with a one-step closure. Patients with severe hypoplasia and a final portal pressure of over 25 mmHg are likely candidates for a 2-step shunt closure or liver transplantation. In the previous report, patients with severe IHPS hypoplasia and a final portal pressure of less than 25 mmHg who were treated with a 1-step closure suffered from decreased portal flow with protracted portal hypertension after the shunt closure. In these cases, the low portal pressure in severe type IHPS hypoplasia may result from the large size of collateral veins leading to reduce the portal pressure under a shunt occlusion test [9]. Hence, we suggest a 2-step closure in all patients with severe hypoplasia of the IHPS in order to avoid associated complications.

In general, an endovascular closure is considered as the first-line treatment because of its less invasiveness compared with surgery [8,9]. A percutaneous endovascular method is preferably selected when the shunt is long enough to accommodate a fixed embolization device without risk of comprised blood flow to the liver and other organs. If so, a surgical closure is considered as a more appropriate method—like in our case. Furthermore, patients with an unacceptable portal pressure after an occlusion test or with extreme hypoplasia of the intrahepatic portal branches are more suitable for stage closure by surgical technique [1,8,10].

The principle steps of the surgical closure include (1) insertion of a catheter into the portal system; (2) isolation and temporary occlusion of the shunt, the change in venous pressure before and after shunt occlusion is recorded; (3) evaluation of the intrahepatic portal venous blood flow via Doppler ultrasonography or intraoperative portal venography before and after occlusion; and (4) during the 15-minute shunt occlusion, the small bowel is examined whether it is edematous or cyanotic. If a rise in portal pressure does not exceed 25 mmHg, the appearance of the bowel is not worrisome and there is an accompanying increase in portal venous flow, then the shunt could be permanently ligated. If the previous criteria are not met, an initial procedure is performed to partially occlude the shunt where there is a portal pressure of less than 20 mmHg. Subsequently, when imaging studies show adequate development of intrahepatic portal vein branches, a second procedure will be performed to completely ligate the shunt. This generally occurs several months after the initial procedure [7,10]. The shunt should be occluded as close to the caval system as possible to provide maximal intrahepatic blood flow. Heparin is given before the occlusion and continued for one to several weeks to prevent possible blood clot formation in the blind segment [10].

Postoperative imaging by Doppler ultrasonography, CT or MRI angiogram should be performed to confirm the degeneration of the shunt and the development of intrahepatic portal branches. Careful follow-up is necessary for several years to monitor the resolution of complications, and to detect the presence of any additional shunts which may be required for the closure to be completed successfully [10].

## 4 Conclusion

This case report has delineated the successful detection and management of a young male patient with extrahepatic CPSS. Physicians should consider CPSS in their differential diagnosis of children who present with unexplained portal hypertension and its associated symptoms. Early diagnosis and appropriate management of CPSS will lead to a favorable prognosis and prevent serious complications that may arise.

## Funding

No source of funding.

## Ethical approval

This is a case report study; no ethical approval was needed.

## Consent

The informed consent was obtained from the patient’s mother for publication of this case report.

## Author contribution

Dr. Suthida conceptualized and wrote this paper.

Dr. Chollasak, a main surgeon involved in care of the patient, edited the manuscript.

Dr. Suporn edited the manuscript.

Dr. Nichanan reviewed the radiological findings in this patient.

## Registration of research studies

Not applicable.

## Guarantor

The Guarantor is Dr. Chollasak Thirapattaraphan.

## Provenance and peer review

Not commissioned, externally peer-reviewed.

## Declaration of Competing Interest

No conflict of interests in this article.
